# PET imaging of microglial pathology in multiple sclerosis

**DOI:** 10.1097/WCO.0000000000001490

**Published:** 2026-04-17

**Authors:** Olli Hartiala, Joel Tuomaala, Laura Airas

**Affiliations:** a Neurocenter, Turku University Hospital; b Turku PET Centre, Turku University Hospital, University of Turku and Åbo Akademi University; c Clinical Neurosciences; d InFLAMES Research Flagship, University of Turku, Turku, Finland

**Keywords:** microglia, multiple sclerosis progression, positron emission tomography, translocator protein

## Abstract

**Purpose of review:**

This review evaluates recent advances in the development of translocator protein (TSPO) – and purinergic receptor–binding PET tracers and highlights the capacity of TSPO-PET-imaging to capture microglial activation across multiple regions of interest in multiple sclerosis brain. We discuss the added value of integrating PET-derived measures with fluid and metabolic biomarkers, as well as their successful application in recent clinical trials.

**Recent findings:**

Recent research highlights PET as a robust molecular imaging tool for detecting microglial activation and implicates dysregulated microglial activity as a key driver of smouldering multiple sclerosis pathology. PET-detectable microglial activation appears not merely as a secondary response to neuroaxonal injury but is increasingly recognized as an integral inflammatory component of ongoing pathological processes that lead to future brain atrophy and clinical deterioration.

**Summary:**

Recent advances establish PET as an essential research tool for evaluating the presence of smouldering inflammation in MS brain not detectable using MRI. Furthermore, PET-based methods have proven suitable for measuring glial responses to potentially neuroprotective therapies currently under development.

## INTRODUCTION

Microglial activation is a central component of multiple sclerosis (MS) pathology involved in promoting both lesion evolution and diffuse neuroaxonal injury [1,2]. While conventional MRI is highly sensitive in capturing acute focal inflammatory activity and subsequent chronic lesion scars, it provides limited capacity to capture compartmentalized, chronic glial cell-driven inflammation which is characteristic for the pathology contributing to MS progression independent of relapse activity [3]. In research settings, positron emission tomography (PET) is increasingly used as a complementary imaging modality enabling *in vivo* assessment of glial pathology by utilization of radiotracers binding to molecules expressed in activated glia. Owing to the capacity to provide molecular-level specificity and the possibility to quantify the molecular targets in living human brain, PET imaging can be viewed as an ideal method to study the neuropathological processes in MS. Importantly, unlike in a conventional neuropathology setting, PET imaging can be performed longitudinally, and can thus provide dynamic insights of glial pathology otherwise not obtainable [4^▪^]. 

**Box 1:**
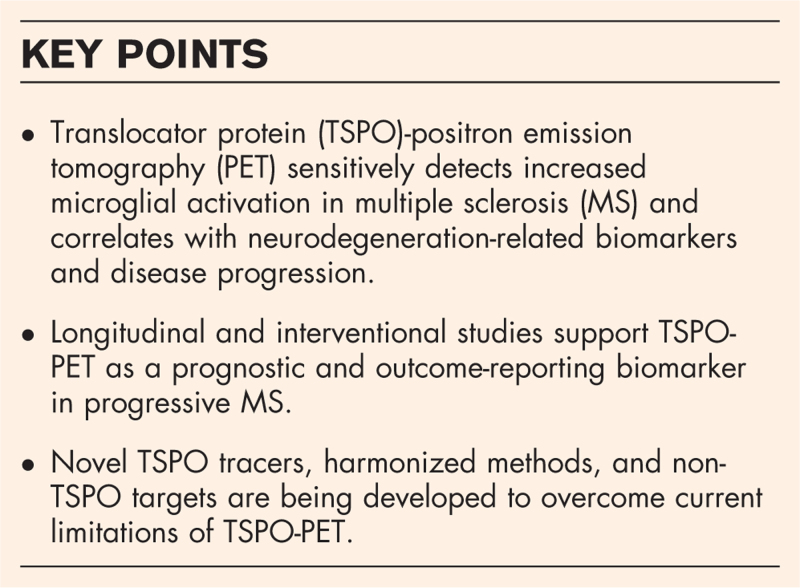
no caption available

**FIGURE 1:**
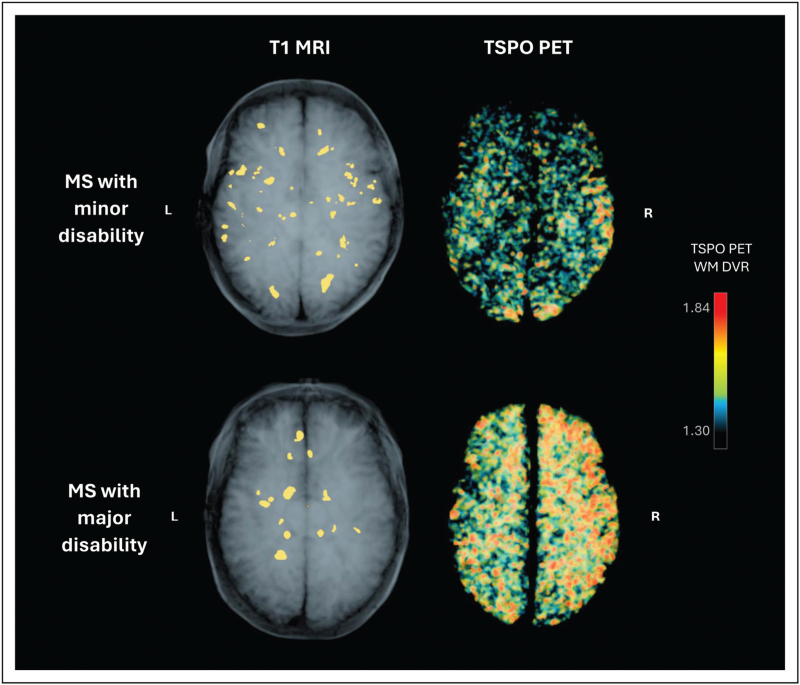
Lesion locations and areas of high microglial activation in the white matter of female MS patients with either minor disability (EDSS 1.0, disease duration 9.6 years) or major disability (EDSS 6.0, disease duration 7.6 years). Microglial activation, as measured by TSPO distribution volume ratio (DVR), extends beyond focal MRI lesions and reveals smouldering inflammatory activity invisible to MRI particularly in the patient with major disability. Volumetric MRI and white matter (WM) TSPO PET images are rendered with transparency to visualize lesion locations and TSPO availability throughout the WM volume. T1 MRI image is overlayed with MS lesion locations.

Most clinical PET studies in MS have focused on the assessment of the mitochondrial 18-kDa translocator protein (TSPO), which is upregulated in activated microglia and to a lesser extent in astrocytes [5]. Experimental evidence indicates that TSPO is involved in mitochondrial cholesterol transport, regulation of reactive oxygen species, calcium signalling and modulation of the mitochondrial permeability transition pore, linking it to cellular stress responses [6] but TSPO may also be functional in regulation of mitochondrial energy metabolism [7]. In chronic neurological conditions such as progressive MS, microglial cells, when activated, tend to get arrested in a proinflammatory, neurodegenerative state [8]. They congregate in areas of ongoing central nervous system (CNS) pathology, and the increased densities of the TSPO-expressing cells can be readily detected using PET and TSPO-binding radioligands, where they manifest as hot areas of increased radioligand binding (Fig. 1) [9]. Consequently, TSPO has become a robust target for imaging MS neuropathological activity [10]. Though PET tracers for TSPO have been utilized since 1984 [11], the continued development of TSPO-PET quantitation methodology, the ever increasing TSPO-PET-studied MS patient numbers and the possibility to combine TSPO-PET outcomes with other imaging and soluble biomarker outcomes in a multimodal fashion has proven uniquely informative for the understanding of the significance of the chronic and focal glial activation that characterize the MS disease [12].

TSPO-PET imaging has consistently demonstrated increased signal not only in the context of focal chronic lesions but also in the normal-appearing white matter (NAWM) and grey matter, supporting the concept of widespread innate immune cell activation beyond MRI-visible focal pathology.

Recently published work has emphasized longitudinal change in TSPO-signal and its’ associations with disability and neurodegeneration. Significant correlations with TSPO-binding and MS pathology-relevant soluble biomarkers measurable in blood have been demonstrated. This emerging work positions TSPO-PET as a specific and quantitative imaging biomarker of MS progression-related pathology. This review focuses on literature from 2024 to 2025, detailing how PET imaging of glial activity is evolving into a predictive biomarker for disease trajectory and therapeutic response.

## PET REVEALS CHRONIC NEUROINFLAMMATION

Earlier abundant work has demonstrated that TSPO-binding in the NAWM is higher in MS patients compared to healthy controls, in males vs. females, and is associated with clinical and radiological measures of disease burden [13–16].

### PET imaging outcomes addressing central nervous system pathology in multiple sclerosis

Using the third-generation TSPO radioligand ^11^C-ER176 PET, Zeydan *et al.* found that PET signal was highest in PPMS patients, followed by patients with RRMS and lowest in controls, and that higher thalamus radioligand uptake correlated with increased disability [17^▪^]. Another study utilizing both proton magnetic resonance spectroscopy and ^11^C-ER176 PET found that glial activation in the thalamus correlated with thalamic volume loss and increased disability in MS patients [18]. In a study of 23 MS patients and 13 HCs combining ^11^C-PBR28-PET and DTI-MRI, TSPO expression was significantly associated with white matter (WM) degeneration [19], confirming previous findings [20,21].

In addition to widespread, compartmentalized microglial activation in areas of white and grey matter, lesion-associated microglial activation plays a significant role in promoting MS progression. A novel chronic active lesion phenotype linked to rapid MS progression, a broad rim lesion (BRL), was discovered in a post-mortem study of a large MS autopsy cohort (*N* = 186). Radiological BRLs (rBRLs) could be visualized using TSPO-PET in another cohort of 114 MS patients, where rBRLs similarly associated with more rapid disease progression, thus establishing the PET-detectable rBRLs as a potential imaging biomarker of significantly detrimental MS pathology [22^▪^].

Other imaging methods for chronic active lesions include susceptibility-weighted imaging for paramagnetic rim lesions (PRLs) [23] and identification of slowly expanding lesions (SELs) from consecutive conventional MRI scans using a Jacobian method [24]. Furthermore, TSPO-PET imaging can be used to quantitate microglial activation in chronic lesions. All three imaging methods, i.e. PRLs, SELs, and TSPO-rim-active lesions [25] capture detrimental pathology [26], but their exact respective pathological characteristics remain somewhat unexplored. While a potential correlation of SELs and TSPO-binding is yet to be reported, a study including 30 MS patients and 21 healthy controls utilizing 7 T MRI and ^11^C-PBR28 PET, showed a correlation between the number of PRLs and PET overall-active lesions. However, no association between the number of peripherally active lesions with PET and PRLs was observed and standard uptake value (SUV) ratio means were similar between PRL and non-PRL lesions. Furthermore, PET-detectable inflammation showed the strongest association with neurological disability highlighting its superior sensitivity in revealing compartmentalized inflammation [27].

Beyond the aforementioned brain regions, Herranz *et al.* showed increased ^11^C-PBR28 binding in the meningeal tissue and cortex of 49 MS patients compared to 21 age-matched controls. These findings were further validated using immunohistochemistry of post-mortem secondary progressive MS (SPMS) cases and controls, indicating a role of meningeal inflammation in MS pathology [28].

Finally, in contrast to the abundance of reported positive correlations of TSPO uptake and MS pathology, a study of 5 MS patients and 23 HCs investigating the two isozymes of cyclooxygenase, i.e. COX-1 expressed in microglia and COX-2 localized in neurons and induced by inflammatory stimuli, found no difference in ^11^C-PS13 binding for COX-1 or ^11^C-MC1 binding for COX-2 between MS patients and HCs or between lesions and normal-appearing brain tissue [29].

### Utilization of TSPO-PET imaging for predicting multiple sclerosis progression

There is increasing evidence that microglial activation relates to later downstream neurodegeneration and clinical progression metrics. A study by Nylund *et al.* reported that accumulation of glial activation relates to later brain atrophy on longer follow-up 4–11 years after PET, supporting TSPO-PET as a readout of compartmentalized inflammation that is relevant to neurodegeneration [4^▪^]. In line with this, RRMS patients who converted to SPMS during a 5-year follow-up had increased TSPO binding in the NAWM, thalamus, and perilesional area at baseline compared to those who remained in the RRMS phase [30^▪^].

### Correlation between soluble biomarkers and TSPO-PET imaging outcomes

PET imaging of glial activation is technically challenging and expensive and presently mostly suitable for use in research settings. There is thus a great demand for more easily accessible biomarkers for assessment of aspects of smouldering CNS inflammation particularly in the context of progressive MS. Combining PET imaging with analysis of soluble biomarkers has potential to inform on the nature of the CNS pathology associated with alterations in biomarkers measurable in blood.

Increased serum neurofilament light chain concentration (sNfL) correlated with increased TSPO-PET signal in the NAWM and chronic active lesions, suggesting that microglial activation, when present, contributes to neuroaxonal damage leading to increased sNfL in blood [31]. Blood glial fibrillary acidic protein (GFAP), a marker of astrogliosis, on the other hand demonstrated a positive correlation with a greater volume of lesions harbouring increased TSPO-binding among a mixed cohort of MS patients. This suggests that in this context, GFAP concentration in blood might reflect mixed microglia and astrocyte pathology within chronic lesions, as TSPO is also expressed on a subset of astrocytes in addition to microglial cells. These findings associated with more unfavourable brain volume and overall lesion volume metrics measured by MRI [32]. Furthermore, higher levels of plasma chitinase-3-like protein 1, expressed both by activated astrocytes and microglia, correlated with higher brain TSPO uptake in a cohort of 55 MS patients [33].

### Longitudinal TSPO-PET imaging in multiple sclerosis

A number of studies have addressed the usability of longitudinal TSPO-PET imaging in clinical therapeutic settings, allowing dynamic evaluation of in situ glial activation in living patients with or without treatment [34–36]. Recently, the first publication reporting a longitudinal change in TSPO-binding in an untreated MS cohort was published. Here, SPMS patients had higher baseline TSPO-binding compared to RRMS in the NAWM. Over a one-year follow-up, the radioligand binding increased among SPMS but not RRMS patients [4^▪^]. The findings implicate a gradual increase in glial activation over time in patients prone to progression. The effect of chimeric antigen receptor T-cell (CAR-T) therapy on microglia activity was studied in a small open-label study including 5 MS patients treated with anti-B-cell maturation antigen CAR-T. The study showed a reduction in ^11^C-PBR28 uptake in three of the five patients and no change in the other two [37].

Currently, a number of early-phase intervention studies targeting glial activation and utilizing TSPO-PET imaging as a primary endpoint are on-going. Disease-modifying therapies in these studies include cladribine [38], ocrelizumab [39], ofatumumab [40], foralumab[41], *N*-acetyl cysteine [42], istradefylline [43], and hydroxychloroquine[44].

### Emerging TSPO-PET radioligands

First-, second-, and third-generation TSPO radioligands have proven valuable in imaging microglial activation in MS research, but suffer from compromises in TSPO specificity, sensitivity, low signal-to-noise ratio, brain permeability and clearance kinetics, haplotype affinity and effective dose. ^18^F-FEDAC was developed to match the high TSPO affinity of other second-generation TSPO radioligands while improving on slow clearance of radioactivity from the brain [45]. The first human study with seven healthy participants found ^18^F-FEDAC to have a similar biodistribution to other second-generation TSPO radioligands whilst exhibiting minimal accumulation in healthy brain [46]. Subsequent work in rat stroke models confirmed ^18^F-FEDAC's sensitivity to neuroinflammation-associated increases in TSPO expression [47]. ^18^F-BIBD-239, a novel tracer with reduced polymorphism sensitivity, was recently developed to overcome the short half-life that limits current ^11^C-labeled third-generation radioligands. In the first *in vivo* human study ^18^F-BIBD-239 was shown to rapidly permeate into the healthy brain and to accumulate in inflamed TSPO-expressing glioma tissue [48]. ^18^F- FEDAC and ^18^F-BIBD-239 both demonstrate preliminary suitability as PET imaging biomarkers of microglial activation with potential application in neuroinflammatory conditions such as MS. In addition, ^11^C-DPA-813 and ^18^F-DPA-814 are two other novel third-generation TSPO-PET tracers showing high binding to TSPO on human MS tissues in autoradiography but are also yet to be tested in MS patients [49^▪^].

### Emerging PET microglia markers beyond TSPO

While TSPO robustly associates with microglial activation in the MS brain as demonstrated by transcriptomic and neurohistological evidence [50^▪▪^], TSPO is additionally expressed to a lesser extent by other cell types such as astroglial cells, leading to inherent unspecificity of radioligand signal. Furthermore, TSPO-PET does not differentiate between anti-inflammatory neuroprotective and pro-inflammatory neurotoxic microglial activation. Novel PET targets more closely linked to pro-inflammatory microglial phenotypes are under active investigation, although clinical validation remains limited. Such targets include purinergic receptors such as the P2X7 molecule. In a study using the radioligand ^11^C-SMW139, binding to P2X7 receptor did not differ between MS patients and controls. Differences in tracer binding in NAWM or perilesional area were noted across some demographic measures, namely sex, age, and disease duration as well as MS disease type (new-onset or SPMS) but high variability and tracer limitations related to free fraction and quantification stability potentially hinder utilization of this ligand [51].

CSF1R (colony-stimulating factor-1 receptor) regulates glial differentiation and is expressed in microglia and macrophages. CSF1R transcript and protein expression is elevated in MS patient NAWM, perilesional area and CSF, and its inhibition attenuates detrimental neuroinflammation and demyelination in MS models [52] To improve on the specificity and radiological stability of a previously developed CSF1R PET tracer ^11^C-CPPC, an analogous ^11^C-radioligand, ^11^C-1, was recently developed. It labels CSF1R with higher specificity but had poor brain uptake in live mice and non-human primates, deeming it unsuitable for in vivo CNS PET studies [53]. In contrast, another CSF1R inhibitor, ^18^F-JNJ-CSF1R-1, demonstrated the desirable high brain uptake in both mice and non-human primates [54].

### Methodological improvements

Lack of harmonization across radioligands and quantification methods has hindered execution of multicentre studies applying TSPO-PET imaging in MS. The INFLANET project aims to establish a methodological framework to harmonize quantification and analysis of PET imaging across six centres in France using the second-generation TSPO radiotracer ^18^F-DPA-714 [55]. Extending this framework to include even more study centres that may use different scanners and tracers could facilitate further collaboration, potentially building on a novel blood-free and reference-free method recently introduced by Maccioni *et al.* [56] The supervised clustering algorithm has been implemented as a blood-free method for first-generation (^11^C-PK11195) [57] and some second-generation TSPO radioligands, such as ^11^C-PBR28 [58], ^18^FDPA-714 [59] and most recently, ^11^C-DPA-713 [60^▪▪^].

Other efforts to improve upon existing PET methods include a radiation dosimetry study of the existing radioligand ^18^F-PBR111 [61] and development of a novel logarithmically transformed “glial activity load on PET” score (lnGALP). The score is calculated as the sum of voxel-by-voxel *z* -scores ≥4 based on SUV-measurements of ^11^C-PBR28 radioligand binding in voxels within predefined regions of interest. In a cross-sectional study of 22 MS patients and 8 healthy controls re-addressing the presence of compartmentalized inflammation in MS vs. healthy controls, higher lnGALP scores in cortical grey matter and WM were measured in MS patients compared to healthy controls [62]. The usability and reliability of the lnGALP method still awaits validation against conventional modelling methods [58].

### Other PET targets relevant to multiple sclerosis pathology

Radioligands to evaluate synaptic density, myelin content and glucose metabolism have recently been actively studied in MS. Synaptic density measured with ^11^C-UCB-J PET imaging quantifying the synaptic vesicle glycoprotein 2A was reduced in the cortical grey matter of 10 patients with MS compared to 8 healthy controls, with correlation to cognitive impairment [63^▪^]. Similarly, in a study using the radioligand ^18^F-UCB-H and MRI, PET imaging was far more sensitive in detecting cortical pathology in 31 MS patients compared to MRI [64^▪^]. Also here, the extent of PET-defined areas of cortical synapse pathology was associated with disability and cognitive performance [64^▪^].

Myelin density in MS has been studied using a variety of PET radioligands. The radiolabelled derivative of 4-diaminopyridine, ^18^F-3F4AP that binds to potassium channels in demyelinated axons, showed potential in differentiating MS patients (*n* = 3) from healthy controls (*n* = 3) and lesions with and without axonal damage [65^▪▪^]. A study by Barrios-López *et al.* suggested that amyloid PET for assessing myelin integrity may have potential as a biomarker in MS as they reported higher ^18^F-fluorbetaben uptake in damaged WM compared to NAWM and correlations between SUV in the damaged WM and clinical parameters [66]. Yazdan-Panah *et al.* demonstrated the feasibility of assessing cerebral blood flow and myelin content simultaneously using ^11^C-PiB PET imaging, building on the previously reported correlation of ^11^C-PiB PET with 15O-H_2_O PET perfusion measures in the cortex [67]. Yet another PET tracer for myelin imaging, ^11^C-MeDAS, showed lower uptake in the spinal cord of MS patients vs. HCs and its distribution corresponded with known myelin distribution in the spinal cord. However, its sensitivity in detecting spinal MRI lesions was low [68].

Finally, Brier et al. addressed glucose metabolism in the MS brain with fluorodeoxyglucose PET in combination with MRI and showed increased NAWM glycolysis in MS patients compared HCs and that glycolysis increased with increased disability [69].

## CONCLUDING REMARKS

Collectively, PET imaging of microglial pathology offers a unique window into mechanisms of chronic inflammation in MS and holds promise for improving disease stratification, understanding treatment effects and refining biomarkers of progression. PET imaging is expected to have a significant role in future Phase II proof-of-concept trials targeting glial activation to slow down MS progression. Importantly, unlike MRI measures of atrophy, which represent irreversible tissue loss, PET captures the active inflammatory state behind the blood-brain-barrier, offering a potential therapeutic window to halt progression before permanent neurodegeneration occurs.

## Acknowledgements

*None.*

### Financial support and sponsorship

*Olli Hartiala has received support for congress participation and travel from Novartis. Joel Tuomaala has nothing to disclose. Laura Airas has received honoraria and institutional research grant support from Sanofi and Merck Serono. This work was funded by the InFLAMES Flagship Programme of the Research Council of Finland (decision numbers: 337530, 357910 and 358823), and the State Research Funding (SRF) for university-level health research in Turku University Hospital, Wellbeing Services County of Southwest Finland.*

### Conflicts of interest

*There are no conflicts of interest.*

## REFERENCES AND RECOMMENDED READING

Papers of particular interest, published within the annual period of review, have been highlighted as:

▪ of special interest
▪▪ of outstanding interest

## References

[1] O’Loughlin E Madore C Lassmann H Butovsky O. Microglial phenotypes and functions in multiple sclerosis. Cold Spring Harb Perspect Med 2018; 8:a028993. 29419406 10.1101/cshperspect.a028993 PMC5793738

[2] van den Bosch AMR van der Poel M Fransen NL. Profiling of microglia nodules in multiple sclerosis reveals propensity for lesion formation. Nat Commun 2024; 15:1667. 38396116 10.1038/s41467-024-46068-3 PMC10891081

[3] Kuhlmann T Moccia M Coetzee T. Multiple sclerosis progression: time for a new mechanism-driven framework. Lancet Neurol 2023; 22:78–88. 36410373 10.1016/S1474-4422(22)00289-7 PMC10463558

[4▪] Nylund M Lehto J Matilainen M. Longitudinal accumulation of glial activation measured by TSPO-PET predicts later brain atrophy in multiple sclerosis. J Neuroinflamm 2025; 22:200. 10.1186/s12974-025-03519-y PMC12333212 40775641

[5] Hamzaoui M Garcia J Boffa G. Positron emission tomography with [18F]-DPA-714 unveils a smoldering component in most multiple sclerosis lesions which drives disease progression. Ann Neurol 2023; 94:366–383. 37039158 10.1002/ana.26657

[6] Gatliff J Campanella M. TSPO: kaleidoscopic 18-kDa amid biochemical pharmacology, control and targeting of mitochondria. Biochem J 2016; 473:107–121. 26733718 10.1042/BJ20150899

[7] Gut P. Targeting mitochondrial energy metabolism with TSPO ligands. Biochem Soc Trans 2015; 43:537–542. 26551690 10.1042/BST20150019

[8] Kamma E Lasisi W Libner C. Central nervous system macrophages in progressive multiple sclerosis: relationship to neurodegeneration and therapeutics. J Neuroinflamm 2022; 19:45. 10.1186/s12974-022-02408-y PMC8830034 35144628

[9] Airas L Rissanen E Rinne JO. Imaging neuroinflammation in multiple sclerosis using TSPO-PET. Clin Transl Imaging 2015; 3:461–473. 27331049 10.1007/s40336-015-0147-6 PMC4887541

[10] Airas L Nylund M Rissanen E. Evaluation of microglial activation in multiple sclerosis patients using positron emission tomography. Front Neurol 2018; 9:181. 29632509 10.3389/fneur.2018.00181 PMC5879102

[11] Camsonne R Crouzel C Comar D. Synthesis of N-(11 C) methyl, N-(methyl-1 propyl), (chloro-2 phenyl)-1 isoquinoleine carboxamide-3 (PK 11195): A new ligand for peripheral benzodiazepine receptors. J Labelled Comp Radiopharm 1984; 21:985–991.

[12] Airas L Yong VW. Microglia in multiple sclerosis – pathogenesis and imaging. Curr Opin Neurol 2022; 299–306. 35674072 10.1097/WCO.0000000000001045

[13] Guido G Preziosa P Filippi M Rocca MA. TSPO PET in multiple sclerosis: emerging insights into pathophysiology, prognosis, and treatment monitoring. Mult Scler Relat Disord 2025; 100:106546. 40450829 10.1016/j.msard.2025.106546

[14] Sucksdorff M Matilainen M Tuisku J. Brain TSPO-PET predicts later disease progression independent of relapses in multiple sclerosis. Brain 2020; 143:3318–3330. 33006604 10.1093/brain/awaa275 PMC7719021

[15] Datta G Violante IR Scott G. Translocator positron-emission tomography and magnetic resonance spectroscopic imaging of brain glial cell activation in multiple sclerosis. Mult Scler 2017; 23:1469–1478. 27903933 10.1177/1352458516681504 PMC6383749

[16] Laaksonen S Saraste M Nylund M. Sex-driven variability in TSPO-expressing microglia in MS patients and healthy individuals. Front Neurol 2024; 15:1352116. 38445263 10.3389/fneur.2024.1352116 PMC10913932

[17▪] Zeydan B Neyal N Son J. Microglia positron emission tomography and progression in multiple sclerosis: thalamus on fire. Brain Commun 2025; 7:fcaf141. 40322777 10.1093/braincomms/fcaf141 PMC12046125

[18] Kara F Neyal N Kamykowski MG. Decoding thalamic glial interplay in multiple sclerosis through proton magnetic resonance spectroscopy and positron emission tomography. Int J Mol Sci 2025; 26:8656. 40943577 10.3390/ijms26178656 PMC12428814

[19] Tommasin S Giannì C Treaba CA. The association between white matter chronic inflammation and degeneration in multiple sclerosis: a combined 11C-PBR28 PET-MRI study. Mult Scler Relat Disord 2025; 96: 106350. 40036908 10.1016/j.msard.2025.106350

[20] Bezukladova S Tuisku J Matilainen M. Insights into disseminated MS brain pathology with multimodal diffusion tensor and PET imaging. Neurol Neuroimmunol Neuroinflamm 2020; 7:E691. 32123046 10.1212/NXI.0000000000000691 PMC7136049

[21] Poirion E Tonietto M Lejeune FX. Structural and clinical correlates of a periventricular gradient of neuroinflammation in multiple sclerosis. Neurology 2021; 96:E1865–E1875. 33737372 10.1212/WNL.0000000000011700 PMC8105971

[22▪] Klotz L Smolders J Lehto J. Broad rim lesions are a new pathological and imaging biomarker for rapid disease progression in multiple sclerosis. Nat Med 2025; 31:2016–2026. Epub. 40301560 10.1038/s41591-025-03625-7 PMC12176629

[23] Meaton I Altokhis A Allen CM. Paramagnetic rims are a promising diagnostic imaging biomarker in multiple sclerosis. Mult Scler 2022; 28:2212–2220. 36017870 10.1177/13524585221118677 PMC9679799

[24] Elliott C Wolinsky JS Hauser SL. Slowly expanding/evolving lesions as a magnetic resonance imaging marker of chronic active multiple sclerosis lesions. Mult Scler 2019; 25:1915–1925. 30566027 10.1177/1352458518814117 PMC6876256

[25] Polvinen E Matilainen M Nylund M. TSPO-detectable chronic active lesions predict disease progression in multiple sclerosis. Neurol Neuroimmunol Neuroinflamm 2023; 10:e200133. 37349108 10.1212/NXI.0000000000200133 PMC10291892

[26] Bagnato F Sati P Hemond CC. Imaging chronic active lesions in multiple sclerosis: a consensus statement. Brain 2024; 147: 2913–2933. 38226694 10.1093/brain/awae013 PMC11370808

[27] Treaba CA Herranz E Barletta VT. Phenotyping in vivo chronic inflammation in multiple sclerosis by combined 11C-PBR28 MR-PET and 7T susceptibility-weighted imaging. Mult Scler 2024; 30:1755–1764. 39436837 10.1177/13524585241284157 PMC11742271

[28] Herranz E Treaba CA Barletta VT. Characterization of cortico-meningeal translocator protein expression in multiple sclerosis. Brain 2024; 147:2566–2578. 38289855 10.1093/brain/awae030 PMC11224595

[29] Tang S Harrison DM Bardhoshi A. Cyclooxygenase-1 and cyclooxygenase-2 densities measured using positron emission tomography are not altered in the brains of individuals with stable multiple sclerosis. J Cereb Blood Flow Metab 2025; 45:1417–1427. 40367389 10.1177/0271678X251332490 PMC12078256

[30▪] Laaksonen S Sucksdorff M Vuorimaa A. Predictors of risk of secondary progression in multiple sclerosis. Ther Adv Neurol Disord 2025; 18:17562864251357276. 40951543 10.1177/17562864251357276 PMC12423534

[31] Saraste M Matilainen M Vuorimaa A. Association of serum neurofilament light with microglial activation in multiple sclerosis. J Neurol Neurosurg Psychiatry 2023; 94:698–706. 37130728 10.1136/jnnp-2023-331051 PMC10447382

[32] Sjöros T Saraste M Matilainen M. Serum glial fibrillary acid protein associates with TSPO-expressing lesions in multiple sclerosis brain. Ther Adv Neurol Disord 2025; 18: 10.1177/17562864251352998 PMC12314351 40756531

[33] Ahola V Saraste M Nylund M. Plasma CHI3L1 associates with brain volume loss and glial activation in multiple sclerosis. J Neurol Neurosurg Psychiatry 2025; 96:1053–1060. 40379482 10.1136/jnnp-2025-336063 PMC12573333

[34] Lehto J Nylund M Matilainen M. Longitudinal stability of progression-related microglial activity during teriflunomide treatment in patients with multiple sclerosis. Eur J Neurol 2023; 30:2365–2375. 37154404 10.1111/ene.15834

[35] Sucksdorff M Rissanen E Tuisku J. Evaluation of the effect of fingolimod treatment on microglial activation using serial PET imaging in multiple sclerosis. J Nucl Med 2017; 58:1646–1651. 28336784 10.2967/jnumed.116.183020

[36] Sucksdorff M Tuisku J Matilainen M. Natalizumab treatment reduces microglial activation in the white matter of the MS brain. Neurol Neuroimmunol Neuroinflamm 2019; 6:e574. 31355310 10.1212/NXI.0000000000000574 PMC6624093

[37] Qin C Dong MH Zhou LQ. Anti-BCMA CAR-T therapy in patients with progressive multiple sclerosis. Cell 2025; 188:6414–6423e11. 41101309 10.1016/j.cell.2025.09.020

[38] Effect of Cladribine Treatment on Microglial Activation in the CNS (CLADPET). https://clinicaltrials.gov/study/NCT04239820.

[39] Effect of Ocrelizumab on Brain Innate Immune Microglial Cells Activation in MS Using PET-MRI With 18F-DPA714. https://clinicaltrials.gov/study/NCT03691077. 2022.

[40] 9-Month Observational Follow-Up Study to Assess the Effect of Ofatumumab on Microglia Pathology in MS Patients. https://clinicaltrials.gov/study/NCT04510220.

[41] A Study of Nasal Foralumab in Non-Active Secondary Progressive Multiple Sclerosis Patients. https://clinicaltrials.gov/study/NCT06292923.

[42] Physiological Effects of N-Acetyl Cysteine in Patients With Multiple Sclerosis. https://clinicaltrials.gov/study/NCT03032601.

[43] A clinical proof-of-concept study to reduce smoldering inflammation in progressive MS. https://euclinicaltrials.eu/ctis-public/view/2024-517336-21-00.

[44] Hydroxychloroquine in progressive MS. https://ctis.eu/search/trial/2025-522573-11-00.html.

[45] Yanamoto K Kumata K Yamasaki T. [18F]FEAC and [18F]FEDAC: two novel positron emission tomography ligands for peripheral-type benzodiazepine receptor in the brain. Bioorg Med Chem Lett 2009; 19:1707–1710. 19217778 10.1016/j.bmcl.2009.01.093

[46] Tamura K Nishii R Tani K. A first-in-man study of [18F] FEDAC: a novel PET tracer for the 18-kDa translocator protein. Ann Nucl Med 2024; 38:264–271. 38285284 10.1007/s12149-023-01895-0 PMC10954948

[47] Yamasaki T Zhang Y Mori W. Estimation of the binding potential of the PET ligand [18F]FEDAC to translocator protein 18 kDa (TSPO) in the brain of experimental stroke model rats. Nucl Med Biol 2025; 150–151:109579. 10.1016/j.nucmedbio.2025.109579 41207056

[48] Wang X Li X Zheng W. [18F]BIBD-239: a first-in-human PET radiotracer with dual diagnostic utility for glioma grading and myocardial imaging. Mol Pharm 2025; 22:6339–6348. 40970663 10.1021/acs.molpharmaceut.5c01088

[49▪] Beaino W JM Kooijman E Werry EL. Development and evaluation of [11C]DPA-813 and [18F]DPA-814: novel TSPO PET tracers insensitive to human single nucleotide polymorphism rs6971. Eur J Nucl Med Mol Imaging 2025; 52:2658–2670. 39907797 10.1007/s00259-025-07109-1 PMC12119672

[50▪▪] Fagiani F Pedrini E Martire MS. Spatially-restricted inflammation-induced senescent-like glia in multiple sclerosis and patient-derived organoids. Nat Commun 2025; 16:8477. 41006208 10.1038/s41467-025-63371-9 PMC12475462

[51] Lehto J Aarnio R Tuisku J. P2X 7-receptor binding in new-onset and secondary progressive MS – a [11C]SMW139 PET study. EJNMMI Res 2024; 14:123. 39636350 10.1186/s13550-024-01186-3 PMC11621262

[52] Hagan N Kane JL Grover D. CSF1R signaling is a regulator of pathogenesis in progressive MS. Cell Death Dis 2020; 11:904. 33097690 10.1038/s41419-020-03084-7 PMC7584629

[53] Foss CA Naik R Das D. A radioligand for in vitro autoradiography of CSF1R in post-mortem CNS tissues. EJNMMI Res 2024; 14:76. 39186197 10.1186/s13550-024-01133-2 PMC11347546

[54] Salarian M Liu S Tsai H. Evaluation of [18F]JNJ-CSF1R-1 as a positron emission tomography ligand targeting colony-stimulating factor 1 receptor. Mol Imaging Biol 2025; 27:163–172. 40009327 10.1007/s11307-025-01991-9

[55] Study of neuroinflammation in multiple sclerosis by PET-MRI imaging using the radiotracer ([18F]-DPA-714): a multicentre cohort study (INFLANET). https://clinicaltrials.gov/study/NCT06280742.

[56] Maccioni L Brusaferri L Barzon L. A novel blood-free analytical framework for the quantification of neuroinflammatory load from TSPO PET imaging. J Cereb Blood Flow Metab 2025; 45:2283–2300. 40744907 10.1177/0271678X251361261 PMC12316680

[57] Turkheimer FE Edison P Pavese N. Reference and target region modeling of [11C]-(R)-PK11195 brain studies. J Nucl Med 2007; 48:158–167. 17204713

[58] Zanotti-Fregonara P Kreisl WC Innis RB Lyoo CH. Automatic extraction of a reference region for the noninvasive quantification of translocator protein in brain using 11C-PBR28. J Nucl Med 2019; 60:978–984. 30655330 10.2967/jnumed.118.222927 PMC6604696

[59] García-Lorenzo D Lavisse S Leroy C. Validation of an automatic reference region extraction for the quantification of [18F]DPA-714 in dynamic brain PET studies. J Cereb Blood Flow Metab 2018; 38:333–346. 28178885 10.1177/0271678X17692599 PMC5951011

[60▪▪] Lee Y Nguyen TD Du Y. Validating the utility of supervised clustering algorithm for precise [11C]DPA-713 PET brain image quantification. J Nucl Med 2025; 66:764–770. 40180563 10.2967/jnumed.124.268519 PMC12051772

[61] Tournier BB Mansouri Z Salimi Y. Radiation dosimetry of the 18 kDa translocator protein ligand [18F]PBR111 in humans. Nucl Med Biol 2025; 144-145.109011. 40179687 10.1016/j.nucmedbio.2025.109011

[62] Singhal T Cicero S Rissanen E. Glial activity load on pet reveals persistent “smoldering” inflammation in ms despite disease-modifying treatment: 18 F-PBR06 Study. Clin Nucl Med 2024; 49:491–499. 38630948 10.1097/RLU.0000000000005201

[63▪] Luoma A Matilainen M Tuisku JM. Synaptic density in multiple sclerosis: an in vivo study using [11C]UCB-J-PET Imaging. Neurol Neuroimmunol Neuroinflamm 2025; 12:e200435. 40609051 10.1212/NXI.0000000000200435 PMC12227148

[64▪] Ullrich Gavilanes EM Bartos LM Gernert JA. SV2A-PET imaging uncovers cortical synapse loss in multiple sclerosis. Sci Transl Med 2025; 17:eadt5585. 41032626 10.1126/scitranslmed.adt5585

[65▪▪] Tiss A Michaelson NM Russo AW. First evaluation in multiple sclerosis using PET tracer [18F]3F4AP demonstrates heterogeneous binding across lesions. Eur J Nucl Med Mol Imaging 2026; 53:1125–1138. 40759830 10.1007/s00259-025-07454-1 PMC12830403

[66] Barrios-López JM Triviño-Ibáñez EM Piñeiro-Donis A. Baseline findings from dual-phase amyloid PET study in newly diagnosed multiple sclerosis: exploring its potential as a biomarker of myelination and neurodegeneration. J Pers Med 2025; 15:520. 41295222 10.3390/jpm15110520 PMC12653326

[67] Yazdan-Panah A Bodini B Soulier T. Simultaneous assessment of blood flow and myelin content in the brain white matter with dynamic [11C]PiB PET: a test-retest study in healthy controls. EJNMMI Res 2024; 14:50. 38801594 10.1186/s13550-024-01107-4 PMC11130116

[68] van der Weijden CWJ Ahmed AKMA van der Hoorn A. Myelin imaging of the spinal cord in animal models and patients with multiple sclerosis using [11C]MeDAS PET: a translational study. J Nucl Med 2025; 66:136–141. 39638431 10.2967/jnumed.123.266896 PMC11705788

[69] Brier MR Judge B Ying C. Increased white matter aerobic glycolysis in multiple sclerosis. Ann Neurol 2025; 97:766–778. 39714123 10.1002/ana.27165 PMC11890956

